# Motivations for Participation in Parkinson Disease Genetic Research Among Hispanics versus Non-Hispanics

**DOI:** 10.3389/fgene.2019.00658

**Published:** 2019-07-16

**Authors:** Karen Nuytemans, Clara P. Manrique, Aaron Uhlenberg, William K. Scott, Michael L. Cuccaro, Corneliu C. Luca, Carlos Singer, Jeffery M. Vance

**Affiliations:** ^1^John P. Hussman Institute for Human Genomics, University of Miami Miller School of Medicine, Miami, FL, United States; ^2^Dr. John T. Macdonald Foundation Department of Human Genetics, University of Miami Miller School of Medicine, Miami, FL, United States; ^3^Department of Neurology, University of Miami Miller School of Medicine, Miami, FL, United States

**Keywords:** participation, genetics, research, diversity, Parkinson disease

## Abstract

Involvement of participants from different racial and ethnic groups in genomic research is vital to reducing health disparities in the precision medicine era. Racial and ethnically diverse populations are underrepresented in current genomic research, creating bias in result interpretation. Limited information is available to support motivations or barriers of these groups to participate in genomic research for late-onset, neurodegenerative disorders. To evaluate willingness for research participation, we compared motivations for participation in genetic studies among 113 Parkinson disease (PD) patients and 49 caregivers visiting the Movement Disorders clinic at the University of Miami. Hispanics and non-Hispanics were equally motivated to participate in genetic research for PD. However, Hispanic patients were less likely to be influenced by the promise of scientific advancements (*N* = 0.01). This lack of scientific interest, but not other motivations, was found to be likely confounded by lower levels of obtained education (*N* = 0.001). Overall, these results suggest that underrepresentation of Hispanics in genetic research may be partly due to reduced invitations to these studies.

## Introduction

Disproportionate participation in genomic studies across different racial and ethnic groups has significant long-term implications for translational benefits associated with this research. Research that focuses on a limited pool of racial and ethnic diverse populations versus a broader array of populations may lead to potential biases in genomic research findings and restrict benefit to the limited population group (Bustamante et al., 2011). Often the same groups that are underserved in health care are underrepresented in genomic research, thus increasing the potential for future overall health disparities. With the development of a precision care model for health services, more genomic information will be integrated into health services (Biesecker and Green, 2014). This development emphasizes the importance of racial or ethnic specific genetic information with respect to disease risk. Our understanding of population-specific genomic information relies on the inclusion of all racial and ethnic groups in genomic research. The absence of such information will render the implementation of precision medicine in the understudied groups less effective at best. Recent summary studies on genomic reports confirm that approximately 80% of individuals included in genome-wide association studies are European or of European descent, with only 1% Hispanic representation (Bustamante et al., 2011; Popejoy and Fullerton, 2016; Sirugo et al., 2019). Even applications of the more recent next-generation sequencing technology still include over 60% European (descent) individuals (Bustamante et al., 2011; Popejoy and Fullerton, 2016; Sirugo et al., 2019). A common belief is that underrepresentation of racial and ethnic groups in biomedical research is the result of reduced willingness to participate because of mistrust and stigma (Shavers et al., 2002; George et al., 2014; Erves et al., 2017). An alternative position is that underrepresentation can also be ascribed to limited access to research opportunities and reduced invitations to participate (Wendler et al., 2006; Ceballos et al., 2014). Previous studies focusing on participation in research in general have found that among non-white populations, willingness to participate is closely linked to and motivated by concern for personal or overall family or community health (Sanderson et al., 2013; Ulrich et al., 2013; Ceballos et al., 2014; George et al., 2014). Unwillingness, in contrast, is often driven by negative perception of research, lack of personal benefit, and/or fear of results (Sanderson et al., 2013; George et al., 2014; Erves et al., 2017).

Little information is known on the perceived barriers and motivations of patients with late-onset neurodegenerative diseases to participate in research. Despite the higher prevalence of Parkinson disease (PD) and Alzheimer disease (AD) in Hispanics versus white non-Hispanics, reports discussing race or ethnicity in AD or PD health care provide evidence of disparities in prescribing medications (Hemming et al., 2011; Thorpe et al., 2016), referrals to clinical trials (Schneider et al., 2009), availability of resources (Graham-Phillips et al., 2016), and cost (Gilligan et al., 2013) (benefitting non-Hispanic whites more than any other group). Specifically for PD, disparities relating to referrals to deep brain stimulation surgery have also already observed (Chan et al., 2014). This disparity for these disorders extends to genomic research as there is little data on genetic variation (variants, frequency and/or effect size) contributing to PD (or AD) in non-whites. Interestingly, variants unique to a specific racial or ethnic background are reported for PD (e.g., *PINK1* in Asians; Nuytemans et al., 2010) as well as AD (e.g., *ABCA7* frameshift deletion in African Americans; Farrer et al., 1997; Collins, 1999; Calderon et al., 2006; Reitz et al., 2013; Cukier et al., 2016; Feliciano et al., 2016), indicating a clear need to increase research in non-white populations.

Hispanics can harbor variable levels of admixture of European, African, and Native American ancestry in their genetic background (Mao et al., 2007; Price et al., 2007; Bryc et al., 2010). Detailed analyses in the Hispanic population and other admixed populations can thus inform on genetic contributions of disease in the others. Therefore, these groups can be highly instructive in our understanding of genetic disease across race and ethnicity. For example, through analyses of local ancestry, Dr. Rajabli et al. found different risk effects associated with APOEε4 in Hispanic AD depending on the ancestral origin of the region the ε4 was located on (European OR = 10 versus African OR = 3; Rajabli et al., 2018), consistent with previously observed lower APOEε4 risk for AD in an admixed population of African Americans (Farrer et al., 1997). Additionally, after identifying a strong risk effect in African Americans for *ABCA7* (Reitz et al., 2013) (similar to *APOE* in WNH), we recently identified a pathogenic 44-bp deletion in *ABCA7* specific to the African American population and Caribbean Hispanics with an African ancestral background in the *ABCA7* region (Cukier et al., 2016).

To date, despite theirs being the largest minority group in the US (U.S. Census Bureau American Community Survey, 2017), only a handful of genomic studies studying the major PD genes (*LRRK2*, *PARK2*, *PARK7*, *PINK1*, and *SNCA*) have focused on PD patients of Hispanic ancestry (Deng et al., 2006; Alcalay et al., 2010; Marder et al., 2010; Saunders-Pullman et al., 2011; Gatto et al., 2013; Duque et al., 2015; Cornejo-Olivas et al., 2017). These studies often present data in a small sample size of Hispanic patients and summarize across all Hispanic PD patients, regardless of ancestry. Given the high variability of admixture in these populations, caution is warranted for the interpretation and extrapolation of these results. The only study of a large cohort of Hispanic patients (*N* = 1,150) originating from southern South America reports highly variable contribution of LRRK2 p.G2019S (originally observed in European patients) to PD in different Latin American countries (Mata et al., 2011). Additionally, Mata et al. observed an enrichment of an LRRK2 variant p.Q1111H in Peruvian and Chilean, but not Uruguayan or Argentinian PD patients (Mata et al., 2011), suggesting that this variant originated from the Native American genetic background in these patients. Follow-up analyses showed that this variant is common on Native American background and not contributing to disease (Cornejo-Olivas et al., 2017). Alternatively, when screening GBA, a population-specific variant (p.K198E) contributing to disease was only found in the Colombian population (Velez-Pardo et al., 2019). Taken together, the data presented above underscore the need to include admixed and non-European populations in biomedical research of PD and other neurodegenerative disorders to further our understanding of genetic contribution to PD in these populations with complex genetic architectures as well as across all populations (i.e., transethnic).

Here, we wished to evaluate the willingness of patients affected by a late-onset, complex disease (PD) and their caregivers to participate in genomic research, and the main drivers of this willingness across race and ethnicity to potentially identify issues to address and adjust current enrollment protocols to improve participation across all populations.

## Material and Methods

### Human Subject Research Compliance

The presented study was approved by the Institutional Review Board at the University of Miami, and informed consent for the survey was obtained from all participants.

### Participants and Enrollment

All patients were seen by physicians specializing in movement disorders (CS, CCL) at the University of Miami (UM) Health System’s Division of Parkinson’s Disease and Movement Disorders clinic. This division serves as the premier referral center for movement disorders patients from abroad with a particular connection to Latin America and the Caribbean. Both Dr. Singer and Dr. Luca speak Spanish and can address the patient in their preferred language. Summary data from the UM Health System suggest that approximately 35% of PD patients identify as Hispanic. Individuals were eligible for this study if they a) had a clinical diagnosis of PD or b) were caregivers of a person with PD and c) were 18 years of age or older. All eligible individuals were referred to the study by their physicians. Patients who agreed to contribute to this survey were approached about a proposed, hypothetical PD genetic research study closely resembling the one ongoing at the John P. Hussman Institute for Human Genomics at UM. All interviewees received the same information including description of the study purpose and requirements (e.g., a single blood draw, collection of personal and family medical history, and no return of personal results). They were then asked whether they were willing to participate in such a study and to complete a brief survey to indicate the reasons for their decision (i.e., participate vs. not participate). All interactions with participants were conducted in the preferred language of the participant.

### Survey Items

Adapting from a prior in-house study (Cuccaro et al., 2014), we constructed a multi-item survey to assess influences on willingness to participate in genomic/genetic research. This survey asked participants to select reasons that influenced their decision to (refuse to) participate in the proposed genetic study of PD. Individuals who agreed to participate were asked to select from six predefined reasons that could have influenced their decision (e.g., “I want to help find a cure for PD” or “I want to help improve science and knowledge on PD”) (**Table 1**, **Supplementary Table**). Individuals who declined participation were asked to select from 10 reasons for this decision (e.g., “I don’t like having my blood drawn,” “I am concerned my insurance company will find out my results,” or “I don’t trust what will happen with my sample”) (**Supplementary Table**). In addition, we collected socio-demographic information including age, sex, race/ethnicity, and education level. To assess race/ethnicity, we asked participants to indicate what race they identify with, as well as to describe themselves as Latino (indicating geographical ancestral origin in Latin America), Hispanic (referring to Spanish-speaking populations in Latin America with ancestral origin in the Iberian Peninsula), neither, or unknown, and indicate country/region of ancestral origin if known (questions available in **Supplementary Data**).

**Table 1:** Comparison of endorsement rates per reason in Hispanics versus non-Hispanics in patient and caregiver groups.

	Patients			Caregiver		
	WH	WNH	*p*-value*	WH	WNH	*p*-value*
N	72	34		29	18	
Mean age	66.7	67.1		59.3	59.9	
Higher education**, *N* (%)	44 (61.1)	31 (91.2)	0.01	17 (58.6)	16 (88.9)	0.05
Willing to participate, *N* (%)	70 (97.2)	33 (97.1)	1	25 (86.2)	15 (83.3)	1
Motivations selected by those willing to participate, *N* (%)***						
I suffer from PD/have a relative who suffers from PD	59 (84.3)	27 (81.8)	0.78	23 (92.0)	12 (80.0)	0.34
To help future generations with PD	34 (48.6)	20 (62.5)	0.29	11 (44.0)	13 (86.7)	0.01
To help find a cure for PD	66 (94.2)	30 (93.8)	0.68	25 (100.0)	12 (80.0)	0.05
To find new/better treatments for PD	43 (61.4)	28 (87.5)	0.02	17 (68.0)	12 (80.0)	0.49
To improve science and knowledge about PD	32 (45.7)	24 (75.0)	0.01	15 (60.0)	11 (73.3)	0.50
I’m encouraged by family/friend to participate	7 (10.0)	10 (30.3)	0.04	3 (12.0)	4 (26.7)	0.39

*Fisher’s exact test; **Higher education defined as education received after high school; ***Other (encompassing any other reasons participants indicated as motivation, not in the predetermined list) was not analyzed due to low number of endorsements.

### Data Analysis

As described below, we restricted our analyses to individuals who agreed to participate in the proposed, hypothetical genetic study to PD. We tested whether the frequency of endorsement for each of the six reasons for participation differed based on ethnicity using Fisher’s exact tests. Given that only 12 individuals indicated that they would not participate in the proposed genetic study, we did not include these data in the statistical analyses.

## Results

### Participant Description

Over the course of 27 clinic days (1 day a week from November to July), we interviewed 162 individuals, of which 113 were PD patients. The remaining 49 individuals presented themselves as caregivers for the patient (35 spouses/partners, 11 children, 3 other). The majority of patients and caregivers identified as white, Hispanic (WH; ∼63% and ∼59%, respectively) or white, non-Hispanic (WNH; ∼30% and ∼37%). Most of the Hispanic participants were of Cuban ancestry (∼60%), followed by Colombian (∼10%) and Puerto Rican (∼9%) ancestry. These figures correspond to the demographic figures for the larger Miami area. Among remaining participants, 3% of individuals identified as Black/African American, 1.2% identified as Arab, and less than 1% identified as Asian. Given these small numbers, we restricted our analyses to WH (*N* = 101) and WNH (*N* = 52) participants.

Mean age at interview as a function of ethnicity did not differ within the patient (WH 66.7y/WNH 67.1y) or caregiver (WH 59.3y/WNH 59.9y) groups. However, a significant difference in reported education level was observed, with >88% of WNH holding a degree of higher education than high school versus ∼60% in the WH participant group (*p*∼0.0001 across combined patient/caregiver group, **Table 1**).

### Survey Results

Overall, ∼91% of WH and WNH participants reported they would participate in the proposed PD genomic study. Specifically, 97% of the 106 patients would agree to participate, with no observed difference between the two ethnic groups (*p* = 1.00). Among patients, “*To help find a cure for PD*” and “*I suffer from PD*” were endorsed at similarly high frequencies between ethnic groups (∼91%, *p* = 0.68 and ∼83%, *p* = 0.78, respectively), making them the most frequently endorsed reasons for participating in the proposed study (**Table 1**). In contrast, nominally significant higher frequencies of WNH versus WH patients endorsed reasons driven by scientific discovery; “*To find new/better treatments for PD*” (87.5% vs 61.4%, respectively; *p* = 0.02), and “*To improve science and knowledge about PD*” (75% vs 46%, respectively; *p* = 0.01). Additionally, we observed a nominally significant difference for “*I’m encouraged by family/friend to participate*” (WNH 30% vs WH 10%; *p* = 0.04).

In the caregivers group, no difference in willingness to participate in genetic studies was observed, though overall percentage was lower than in patients (86% in WH versus 83% in WNH). Interestingly, while a similar pattern of results was observed among WNH and WH caregivers for the same statements as for the patients, one exception was noted as 86.7% of WNH caregivers endorsed “*To help future generations with PD*” as a reason for participating in the proposed study versus only 44% of WH caregivers (*p* = 0.01).

Given the observed differences in level of education between the two groups, we also analyzed the data based on education status regardless of ethnicity to assess confounding effects (**Table 2**). We observed a nominally significant difference for motivation by “*To improve science and knowledge about PD*” for higher educated versus non-higher educated participants in the patient group (*p* = 0.001; 62.5% vs 26.7%) as well as overall (*p* = 0.002; 64.6% vs 34.9%). No difference in motivation was observed for the caregiver group based on education level.

**Table 2:** Comparison of endorsement rates per reason in higher educated versus non-higher educated participants.

	Patients			Caregiver		Overall	
	Yes	No	*p*-value*	Yes	No	*p*-value*	*p*-value*
Higher education**	74	32		33	14		
Willing to participate, *N* (%)	72 (97.3)	30 (93.7)	0.58	27 (81.8)	13 (92.9)	0.66	1
Motivations selected by those willing to participate, *N* (%)***							
I suffer from PD/have a relative who suffers from PD	59 (81.9)	25 (83.3)	1	23 (85.2)	12 (92.3)	1	0.81
To help future generations with PD	39 (54.2)	14 (46.7)	0.52	17 (63.0)	7 (53.8)	0.73	0.46
To help find a cure for PD	69 (95.8)	28 (93.3)	1	25 (92.6)	12 (92.3)	1	1
To find new/better treatments for PD	51 (70.8)	19 (63.3)	0.49	22 (81.5)	7 (53.8)	0.13	0.16
To improve science and knowledge about PD	45 (62.5)	8 (26.7)	0.001	19 (70.4)	7 (53.8)	0.48	0.002
I’m encouraged by family/friend to participate	11 (15.2)	5 (16.7)	1	7 (25.9)	0 (0.0)	0.07	0.46

*Fisher’s exact test; **Higher education defined as education received after high school; ***Other (encompassing any other reasons participants indicated as motivation, not in the predetermined list) was not analyzed due to low number of endorsements.

## Discussion

Given the growing impact of genomic information on clinical care for increasing numbers of conditions, it is of utmost importance to recognize the genetic differences among racial and ethnic groups. Available research findings for PD or other neurodegenerative disorders on mostly WNH or Asian population groups are not necessarily generalizable to all individuals (Bustamante et al., 2011). Very recently, genetic research for the more common neurodegenerative disease AD in diverse populations of African Americans and Hispanics has shown the power of these analyses across race and ethnicity to identify variants contributing to disease and improve the field’s understanding of disease mechanisms [e.g., GBA (p.K198E) in Hispanics (Velez-Pardo et al., 2019), ABCA7 in Africans and African Americans (Reitz et al., 2013; Cukier et al., 2016), differential risk of APOEe4 on different background (Rajabli et al., 2018)]. These data support the importance to extend genomic research to diverse populations for neurodegenerative disease to fully understand genetic risk factors contributing to disease.

More recently, the number of studies evaluating recruitment issues and methods in different racial and ethnic groups versus the traditional European research population has grown with the rise of precision medicine initiatives, though there are very few for complex, late-onset diseases (Zhou et al., 2016; Hughes et al., 2017). Our results indicate that WH individuals affected by or caring for someone with PD seen at the UM Movement Disorders clinic would be equally willing to participate in genomic research for late-onset disease PD as WNH individuals, given current enrollment protocols. One could argue that the high rate of willingness reflects an increase in interest in research in those individuals seeking treatment at an academic medical center. However, we have observed a significant difference between WNH and WH participants driven by research progress to participate, indicating interest in research has at the very least less priority than other, more personal reasons for WH participants. Additional analyses showed that the lack of motivation of scientific improvement is likely correlated with lower educational level. This divergence could potentially be explained by an underlying lower level of knowledge of or familiarity with basic science and medical research in the WH participant group. Interestingly, the few participants who provided an open answer as reason to participate (“*other*”) indicated they are more willing to participate to help their doctor with whom they have a good relationship. Though these were limited numbers, these data might suggest a higher level of trust between physicians/researchers and participants through a more personal relationship and being helped in the participant’s language of choice.

Taking together the high willingness seen here but current underrepresentation in medical research of WH participants, we offer that the underrepresentation of WH individuals in PD research is in part due to a reduced invitation to participate. It is therefore important for the medical and scientific fields to make a concerted effort to reach out to the different communities and truly establish a relationship as well as inform on and extend participation in (PD) studies to all races and ethnicities. This investment in community outreach will lead to a more equal representation in research and ultimately to a reduction in health disparities.

## Ethics Statement

The presented study was approved by the Institutional Review Board at the University of Miami and informed consent for the survey was obtained from all participants.

## Author Contributions

KN, MC, WS, CS, and CL contributed conception and design of the study; KN, CM, and AU managed and organized the project; KN performed the statistical analysis; KN and MC wrote the first draft of the manuscript; WS, JV, CS, and CL critically reviewed the manuscript. All authors contributed to manuscript revision and read and approved the submitted version.

## Funding

This research was funded by a National Parkinson Foundation Moving Day® grant (PI Nuytemans).

## Conflict of Interest Statement

The authors declare that the research was conducted in the absence of any commercial or financial relationships that could be construed as a potential conflict of interest.

The handling editor declared a past co-authorship with one of the authors WS.
