# Supplementary material for: Motivations for Participation in Parkinson Disease Genetic Research Among Hispanics versus Non-Hispanics
Source: Front Genet. 2019 Jul 16;10:658. doi: 10.3389/fgene.2019.00658 (PMC6646686; doi:10.3389/fgene.2019.00658)
Supplement: Supplementary file 1 [file Table_1.docx]

Supplementary Material

Motivations for participation in Parkinson disease genetic research among Hispanics versus non-Hispanics

**Karen Nuytemans*, Clara P. Manrique, Aaron Uhlenberg, William K. Scott, Michael L. Cuccaro, Corneliu C. Luca, Carlos Singer, Jeffery M. Vance**

*** Correspondence:** Corresponding Author: knuytemans@med.miami.edu

# Supplementary Table

| Motivation | Hesitation |
| --- | --- |
| I suffer from PD/ have a relative who suffers from PD | I do not think participating will benefit me personally |
| To help future generations with PD | I am worried someone will find out my information |
| To help find a cure for PD | I am concerned by insurance company will find out my results |
| To find new/better treatments for PD | I do not trust what will happen with my sample |
| To improve science and knowledge about PD | I did not receive enough information to understand the research |
| I’m encouraged by family/friend to participate | I am not interested |
| Other | I do not like blood draws |
|  | I do not have time |
|  | I do not want to participate for religious reasons |
|  | Other |

**Supplemental Table 1.** Predefined statements provided to participants to support their decision to (not) participate in the proposed PD study

# Supplementary Data

What race do you consider yourself to be? Please select one or more of these categories.

| - American Indian/Alaska Native - Asian - Native Hawaiian or Other Pacific Islander - Black or African American | - White - Other Race - Don't know |
| --- | --- |

You selected "Black or African American", please tell us which group or groups best represents you?

| - African-American - East African - Haitian - Jamaican - North African | - South African - West African - Other Black or African - Don't know |
| --- | --- |

You selected "White". Please indicate which group represents you?

| - Australian/New Zealand - Eastern European (includes Belarus, Bulgaria, Czech Republic, Hungary, Poland, Romania, Slovakia, and Ukraine) - Hispanic/Latino/Spanish (includes Caribbean, Central America, Mexican-American, Mexico, Portugal, South America, Spain, and USA/Canada) - Northern European/Scandinavian (includes Denmark, Estonia, Latvia, Lithuania, Norway, and Sweden) - Russian - Southern European/Mediterranean (includes | Cyprus, Greece, Italy, and Turkey)   - Western European (includes Austria, Belgium, France, Germany, Great Britain, Ireland, Luxembourg, The Netherlands, and Switzerland) - USA/Canada (non-Hispanic/Latino) - Unique Populations (includes Amish, Arab, Finnish, French Canadian, Icelandic, Jewish, Pennsylvania Dutch, etc) (follow-up; which?) - Other White (follow-up; which?) - Don't know |
| --- | --- |

Do you consider yourself Hispanic or Latino?

| - Yes, Latino - Yes, Hispanic | - No - Don't know |
| --- | --- |

Please select the name of the group that represents your Hispanic or Latino origin or ancestry.

| - Spaniard - Portuguese - North American (Mexican/Mexicano/Mexican-American/Chicano) - Caribbean (Cuban/Cuban-American) - Caribbean (Dominican Republic) - Caribbean (Haitian) - Caribbean (Jamaican) - Caribbean (Puerto Rican) - Central American (Belizean) - Central American (Costa Rican) - Central American (Guatemalan) - Central American (Honduran) - Central American (Nicaraguan) | - Central American (Panamanian) - Central American (Salvadoran) - South American (Argentine) - South American (Bolivian) - South American (Brazilian) - South American (Chilean) - South American (Colombian) - South American (Ecuadorian) - South American (Paraguayan) - South American (Peruvian) - South American (Uruguayan) - South American (Venezuelan) - Other Hispanic/Latino (follow-up: which?) - Don't know |
| --- | --- |

**Supplementary Data.** Questions included in survey assessing identification with race/ethnicity of participants.
